# Cell Attachment Capacity and Compounds of Fibrin Membranes Isolated from Fresh Frozen Plasma and Cryoprecipitate

**DOI:** 10.3390/membranes11100783

**Published:** 2021-10-13

**Authors:** Adél Hinsenkamp, Kiara Kun, Fatime Gajnut, Aliz Majer, Zsombor Lacza, István Hornyák

**Affiliations:** 1 Institute of Translational Medicine, Semmelweis University, 1094 Budapest, Hungary; kunkiara1213@gmail.com (K.K.); gajnutfatime@gmail.com (F.G.); majer.aliz@med.semmelweis-univ.hu (A.M.); hornyak.istvan@med.semmelweis-univ.hu (I.H.); 2 Orthosera GmbH, 3500 Krems an der Donau, Austria; zsombor.lacza@orthosera.com; 3 Institute for Sports and Health Sciences, University of Physical Education, 1123 Budapest, Hungary

**Keywords:** fibrinogen, fibrin, cryoprecipitate, mesenchymal stem cells

## Abstract

Fibrin membranes are widely used in regenerative medicine because they are biocompatible, biodegradable, contain growth factors, and support cell attachment. Most commonly they are produced from serum, but they can also be isolated from activated plasma. To increase the fibrinogen concentration of plasma, cryoprecipitate isolation is a possible solution. In this work, cryoprecipitate was prepared from fresh frozen plasma, isolated by plasmapheresis. The concentration of cellular elements, fibrinogen, total protein, and immunoglobulins among others was measured in different concentrations of cryoprecipitates. After activation with Ca-gluconate, fibrin membranes were produced in different thicknesses, and human mesenchymal stem cells were seeded onto the membranes. They were visualized by live-dead staining and their viability was determined by XTT. The platelet-derived growth factor AB content was quantified by ELISA. Our results showed that fibrinogen and platelet concentration can be multiplied in plasma by cryoprecipitate isolation, which affects the thickness and slightly the growth factor content of the membranes. According to live-dead staining, the thickness of the membranes does not influence cell attachment, and XTT measurement did not reveal a significant difference in cell attachment capacity either; however, a growing trend could be observed in the case of some membranes.

## 1. Introduction

Fibrinogen and fibrin are the main factors of hemostasis, angiogenesis, inflammation, wound healing, and other biological and pathological processes. Fibrinogen is a 340 kDa glycoprotein which is normally present in human plasma in 2–5 mg/L concentrations [1]. It is water-soluble, but in the case of vessel injury, or contact with activated blood cells or a foreign surface, a cascade of enzymatic reactions begins [2]. Through a thrombin-mediated proteolytic cleavage, fibrinogen is converted to fibrin. Fibrin polymerization proceeds, and the polymeric fibers are branched enzymatically to form a strong three-dimensional network [3]. Fibrin clots are then dissolved by a fibrinolytic system. Clotting and fibrinolysis are precisely regulated in vivo, as an imbalance towards clotting causes thrombosis [4], while an imbalance towards fibrinolysis leads to bleeding [2].

Cryoprecipitate is a pooled plasma product which can be isolated by thawing frozen plasma at 1–10 °C. The precipitate is rich in fibrinogen, antihemophilic factor (factor VIII), von Willebrand factor, fibronectin, factor XIII, platelet microparticles, and a small amount of immunoglobulins [5], which can be resolubilized in a small volume of plasma [6]. Cryoprecipitate was originally developed as a therapy for treating congenital factor VIII deficiency in the 1950s [7], but later it was also applied in the cases of von Willebrand disease, hypofibrinogenemia, or fibrinogen replacement therapy in fibrinogen deficiency [6]. However, in the case of fibrinogen deficiencies, cryoprecipitate has been largely replaced by human fibrinogen concentrates due to safety reasons [8].

Fibrin is widely used in regenerative medicine, for example as a drug or growth factor delivery system [9], as an adhesive in surgical procedures [10], a sealant in wound healing or regenerative applications [11], for bone repair [12], in oral and maxillofacial surgeries [13], as fibrin glue [14], or as a bioink for 3D bioprinting applicable in hard and soft tissue engineering [15,16].

It can also be applied as a three-dimensional scaffold in tissue engineering [17] because of its biocompatibility, controllable biodegradability, cell attachment promoting properties [18], and growth factor content [19]. Platelet-rich fibrin (PRF) can be isolated from serum immediately after blood drawing into glass tubes [20,21]. Its advantage is that no anticoagulant is added, thus the activation of platelets occurs naturally. However, fibrin membranes can be prepared from the plasma fraction as well. In this case plasma can be isolated from whole blood or by apheresis using anticoagulants, and it can be stored for a longer period of time. The activation of plasma can be achieved by adding thrombin and Ca^2+^ ions [22], or Ca-gluconate [23]. Ca-gluconate is the safer solution because of its non-human and non-animal source.

During blood clotting both in the case of serum- and plasma-based membranes, the platelets are activated and they release their growth factor content. Activated platelets and growth factors are entrapped in the fibrin network [24] and further promote cell attachment to the fibrin membranes and induce the proliferation of adhered cells [25], thus inducing regeneration. Mesenchymal stem cells are the most promising candidates to perform effective regeneration, as they have the ability to differentiate into several lineages, providing novel strategies to improve complex treatments [26].

In this study, fibrinogen, platelet, total protein, and other blood components were measured in cryoprecipitate dissolved in various concentrations in plasma. We aimed to isolate fibrin membranes from cryoprecipitates with different fibrinogen concentrations and determine the ability of human bone marrow-derived mesenchymal stem cells to attach to these membranes. Due to the different solubility of large proteins and the thickness of the membranes, the amount of entrapped growth factors may be different, which affects the cell attachment capacity. Besides, membrane thickness may also have an impact on surgical application, for example when it is important that the scaffold holds the suture [25].

## 2. Materials and Methods

### 2.1. Cryoprecipitate Isolation and Blood Component Measurements

Human fresh frozen plasma (FFP) was purchased from OVSz (Hungarian National Blood Transfusion Service, Budapest, Hungary). It was produced by plasmapheresis, containing citrate as an anticoagulant. The plasma was aliquoted in 50 mL centrifuge tubes, and it was kept at −80 °C for at least 24 h. Cryoprecipitate was isolated according to the following protocol: the plasma was thawed at 3 °C overnight and it was centrifuged at 3260× *g* for 12 min at 3 °C. Cryoprecipitates were isolated in different concentrations, 10, 20, 30, and 40 mL of plasma supernatant was removed, and the precipitate was dissolved in the remaining 40 (C4), 30 (C3), 20 (C2), and 10 (C1) ml of the supernatant, respectively. The samples were kept at 4 °C and they were not frozen to avoid platelet activation.

Samples were taken from the control plasma and from all types of cryoprecipitates and supernatants. Their fibrinogen (*n* = 6), IgG (*n* = 6), IgM (*n* = 6), hemoglobin (*n* = 4), albumin (*n* = 6), and total protein (*n* = 6) concentrations, and ALP enzyme activity (*n* = 4), were measured using a Beckman Coulter AU5800 automated laboratory machine (Brea, CA, USA), and a Sysmex XN-1000 Sa-01 cell counter (Kobe, Japan) was used for the quantitative determination of the platelets (*n* = 5), red blood cells (*n* = 4), and leukocytes (*n* = 4).

### 2.2. Fibrin Membrane Preparation and Weight Measurement

Fibrin membranes were produced from the plasma, cryoprecipitate, and supernatant. 2 mL of the plasma, cryoprecipitate, or supernatant was poured into the wells of a 24-well plate, and 400 µL 10 *w*/*w*% of autoclaved Ca-gluconate solution was added to each well to induce clotting. The plate was kept at 4 °C for 48 h, then the fibrin clot was detached from the wall of the wells, but not from the bottom, using a sterile spatula. The clots were centrifuged in a plate centrifuge at 2020× *g* for 30 min at room temperature to obtain flat fibrin membranes. The membranes were freeze-dried and their weights were measured using an analytical balance.

### 2.3. Human PDGF-AB ELISA

After, cryoprecipitate isolation samples were taken from the cryoprecipitate groups, the control, and the supernatant, and they were stored at −20 °C until ELISA measurement. Venous blood was collected from healthy donors (men and women, 24–45 years of age). Phlebotomy occurred under Institutional Review Board (IRB) approval (IRB approval number: 29152-3/2019/EÜIG). Sodium citrate was used as an anticoagulant, and the plasma was isolated by centrifugation at 1700× *g* until the plasma and red blood cells were separated. The plasma fraction was removed and stored at −20 °C.

The samples were thawed at room temperature and they were activated with CaCl_2_ and human thrombin: 10 mL 1M CaCl_2_ and 10 mL (500 U/mL) human thrombin were added to each 0.5 mL sample. After 30 min the samples were centrifuged at 1000× *g* for 15 min at 25 °C. The supernatant was collected for ELISA measurement.

The concentration of human PDGF-AB (platelet-derived growth factor AB) was measured in the samples using ELISA kit (R&D Systems, Bio-Techne, Minneapolis, MN, USA) according to the manufacturer’s instructions. The FFP samples were derived from four donors, and manually isolated plasma samples were taken from five donors. All samples were measured in duplicate.

### 2.4. Cell Culture

Human bone marrow-derived mesenchymal stem cells (hBM-dMSCs) were cultured in T75 TC treated culture flasks in an incubator at 37 °C, 5% CO_2_, and 95% humidity. The hBM-dMSCs were maintained in a stem cell medium: Dulbecco’s modified Eagle’s medium (DMEM) containing 4.5 g/L glucose and L-glutamine (Lonza, Basel, Switzerland) supplemented with 10% fetal bovine serum (FBS; EuroClone, Pero, Italy), 1% Penicillin– Streptomycin (Sigma-Aldrich, St. Louis, MO, USA), and 0.75 ng/mL basic fibroblast growth factor (Sigma-Aldrich, St. Louis, MO, USA). The culture medium was refreshed 3 times a week, and all cell culture procedures were carried out in a sterile laminar flow tissue culture hood.

### 2.5. Live-Dead Staining of Mesenchymal Stem Cells Cultured on the Fibrin Membranes

The freshly isolated fibrin membranes were placed onto ultra-low attachment 24-well plates in 2 mL of stem cell medium, and 25,000 hBM-dMSCs (5 passages) were seeded onto the membranes and cultured on them for 6 days. The medium was refreshed every 2 days. On the 7th day live-dead staining was performed to visualize attaching cells. The membranes were washed 3 times with PBS (phosphate-buffered saline) and stained in PBS containing 1 mM Calcein-AM (Invitrogen, Carlsbad, CA, USA), 4 mg/mL ethidium homodimer (Invitrogen), and 20 mg/mL Hoechst (Invitrogen, Carlsbad, CA, USA) for 30 min. The gels were washed again 3 times for 10 min with FluoroBrite DMEM (Gibco, Paisley, Scotland), and images were taken by an inverse fluorescent Nikon Eclipse Ti2 microscope (Tokyo, Japan).

### 2.6. Viability of hBM-dMSCs Cultured on the Fibrin Membranes Measured by XTT

The fibrin membranes were placed into the wells of 24-well, ultra-low attachment plates in 500 µL of stem cell medium, and 25,000 hBM-dMSCs (6 passages) were seeded on each membrane. On the next day the viability of the seeded cells was measured using Cell Proliferation Kit II (XTT; Roche, Mannheim, Germany), according to the manufacturer’s instructions, on half of the membrane-containing wells to measure the amount of attached cells. The rest of the membranes with the seeded cells were cultured for 6 more days in a 2 mL stem cell medium. The medium was refreshed every 2 days. On the 7th day the viability of the cells on the membranes was examined using XTT to compare the proliferative effect of the different membranes.

### 2.7. Statistical Analysis

A one-way analysis of variance (ANOVA) with Tukey’s post hoc test was performed to compare the means of groups using Prism 7 software. The significance level was *p* < 0.05, where * means that *p* is between 0.01 and 0.05, ** means that *p* is between 0.01 and 0.001, and *** means that *p* is lower than 0.001, and data are presented as mean ± standard error of the mean.

## 3. Results

### 3.1. Blood Component Measurements in Cryoprecipitates

When frozen plasma is thawed at 1–10 °C and centrifuged, the enrichment of some blood components can be observed in the precipitate due to the reduced dissolution at lower temperatures. During this experiment, the precipitate was resolubilized in altering plasma volumes, and the concentration of different blood components was measured.

Although plasma isolated by plasmapheresis is theoretically free from cellular elements, a small amount of platelets, leukocytes, and red blood cells were found in some of our samples. In control samples, approximately 10 × 10^9^/L platelets were measured compared to manually isolated PPP (platelet-poor plasma), which contains 34.5 × 10^9^/L [27]; however, the platelet count in plasma highly depends on the isolation method, and the normal platelet count of whole blood is 150–450 × 10^9^/L [28].

**Figure 1:**
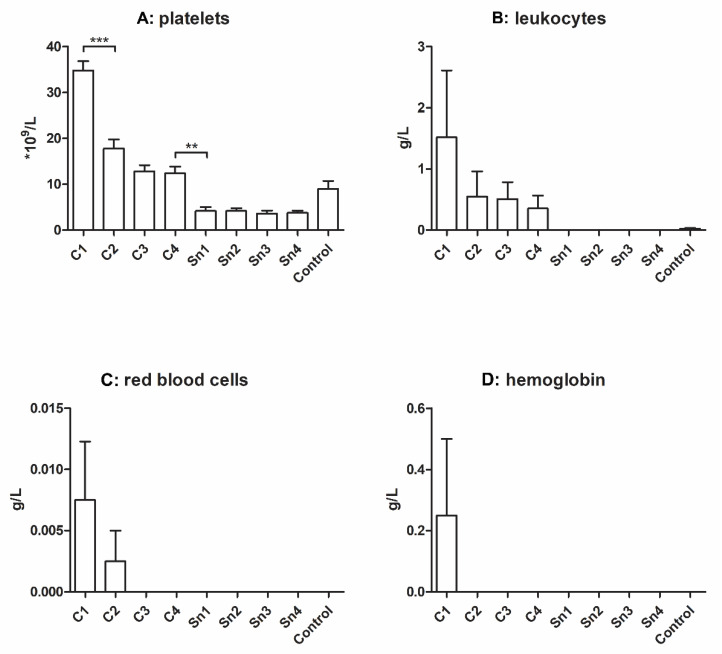
Cellular elements (**A**): platelets (*n* = 5), (**B**): leukocytes (*n* = 4), (**C**): red blood cells (*n* = 4), and (**D**): hemoglobin (*n* = 4) concentration of different cryoprecipitates. The cryoprecipitate was dissolved in 10 mL (C1), 20 mL (C2), 30 mL (C3), and 40 mL (C4) plasma, Sn1, Sn2, Sn3, and Sn4 are the supernatants collected from above the cryoprecipitate, respectively, and plasma was used as a control. The significance level was *p* < 0.05, where * means that *p* is between 0.01 and 0.05, ** means that *p* is between 0.01 and 0.001, and *** means that *p* is lower than 0.001, and data are presented as mean ± standard error of the mean.

The cryoprecipitate was reported to contain platelet microparticles [6] and we observed that whole platelets were also present and more-concentrated cryoprecipitates contained more platelets than less-concentrated cryoprecipitates, control plasma, and supernatants. Significant differences were found between C1 and C2, and between C4 and the supernatants (Figure 1A).

Besides, more leukocytes were found in the more concentrated groups, but the differences were not significant. The control group also contained some leukocytes, but in the supernatants their number was under detectable limits. In the case of red blood cells, we could measure them only in the C1 and C2 groups. The hemoglobin level was examined to obtain information about disrupted red blood cells, but its level was measurable only in the C1 group (Figure 1B–D).

**Figure 2:**
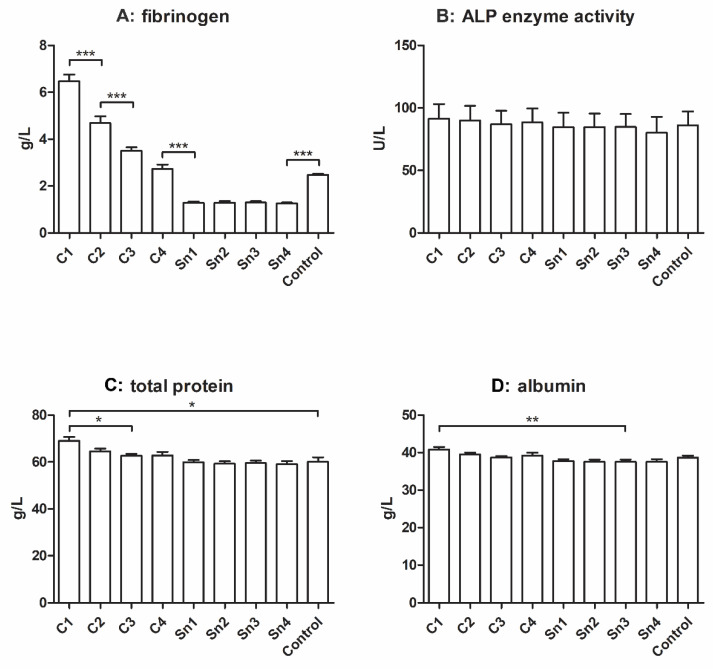
Fibrinogen ((**A**), *n* = 6), total protein ((**C**), *n* = 6), and albumin ((**D**), *n* = 6) concentrations, and ALP enzyme activity measurement ((**B**), *n* = 4). The cryoprecipitate was dissolved in 10 mL (C1), 20 mL (C2), 30 mL (C3), and 40 mL (C4) plasma, Sn1, Sn2, Sn3, and Sn4 are the supernatants collected from above the cryoprecipitate, respectively, and plasma was used as a control. The significance level was *p* < 0.05, where * means that p is between 0.01 and 0.05, ** means that *p* is between 0.01 and 0.001, and *** means that *p* is lower than 0.001, and data are presented as mean ± standard error of the mean.

The main protein component of cryoprecipitate is fibrinogen, and in our research, it was also the most important. In the control sample its concentration was approximately 2.5 g/L, which amount was multiplied in the case of C1 (in average 6.5 g/L). Significant differences were found between C1 and C2, C2 and C3, C4 and the supernatants, and between the supernatants and the control group (Figure 2A).

The ALP (alkaline phosphatase) enzyme catalyzes the dephosphorylation process of proteins, nucleic acids, and small molecules, and can be found in numerous tissues, thus it is often used as a biomarker [29]. Its activity was measured in the cryoprecipitate samples and although no significant difference was observed, it may be seen that it slightly decreases with decreasing cryoprecipitate concentrations (Figure 2B).

The total protein concentration can be observed in Figure 2C, and it was also slightly affected by cryoprecipitate isolation, but a significant difference was only found between C1 and C3 and between C1 and the control, and the supernatants. In the albumin content (Figure 2D), a significant difference was found between C1 and the supernatants; however, the tendency of decreasing albumin concentration with decreasing cryoprecipitate concentration is not clear.

**Figure 3:**
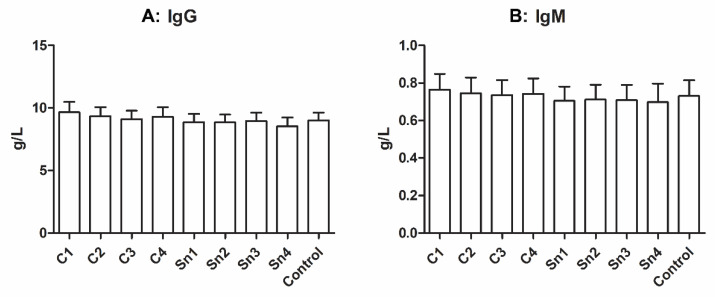
Immunoglobulin concentrations in cryoprecipitate. (**A**): IgG, *n* = 6, (**B**): IgM, *n* = 6. The cryoprecipitate was resolubilized in 10 mL (C1), 20 mL (C2), 30 mL (C3), and 40 mL (C4) plasma, Sn1, Sn2, Sn3, and Sn4 are the supernatants collected from above the cryoprecipitate, respectively, and fresh frozen plasma was used as a control.

It was reported that cryoprecipitate contains a small amount of immunoglobulins [5], thus we measured the IgG and IgM content of our samples. In Figure 3 a faint decreasing tendency might be seen with decreasing cryoprecipitate concentrations both in the case of IgM and IgG; however, no significant difference was observed between the sample groups.

### 3.2. Weight Measurement of the Freeze-Dried Fibrin Membranes

**Figure 4:**
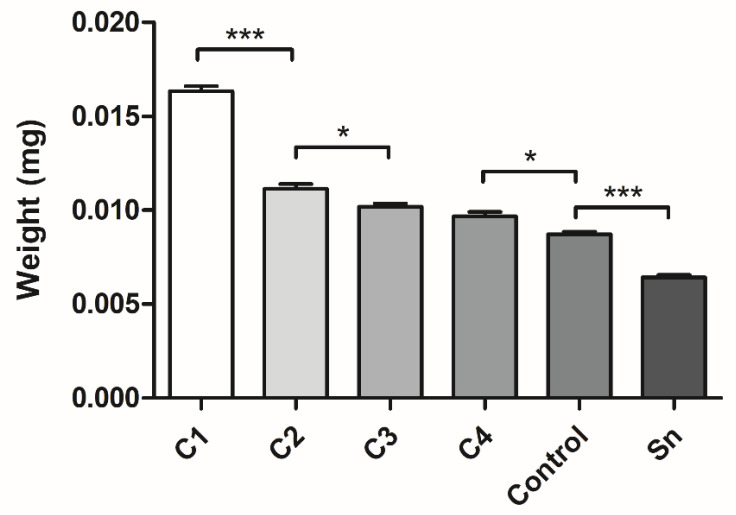
The weights of the freeze-dried different thickness fibrin membranes. The membranes were isolated from cryoprecipitate, which was dissolved in 10 mL (C1), 20 mL (C2), 30 mL (C3), and 40 mL (C4) plasma, from supernatant (Sn), which was collected from above the cryoprecipitates and pooled, and from plasma, which was used as a control (*n* = 4). The significance level was *p* < 0.05, where * means that *p* is between 0.01 and 0.05, ** means that p is between 0.01 and 0.001, and *** means that *p* is lower than 0.001, and data are presented as mean ± standard error of the mean.

After the isolation of the membranes with different thicknesses, they were freeze-dried and their weights were measured using an analytical balance. The measurement supported our expectations, as the thicker membranes’ weights were greater, and significant differences were found between C1 and C2, C2 and C3, C4 and the control, and between the control and supernatants (Figure 4).

### 3.3. Human PDGF-AB Concentration

**Figure 5:**
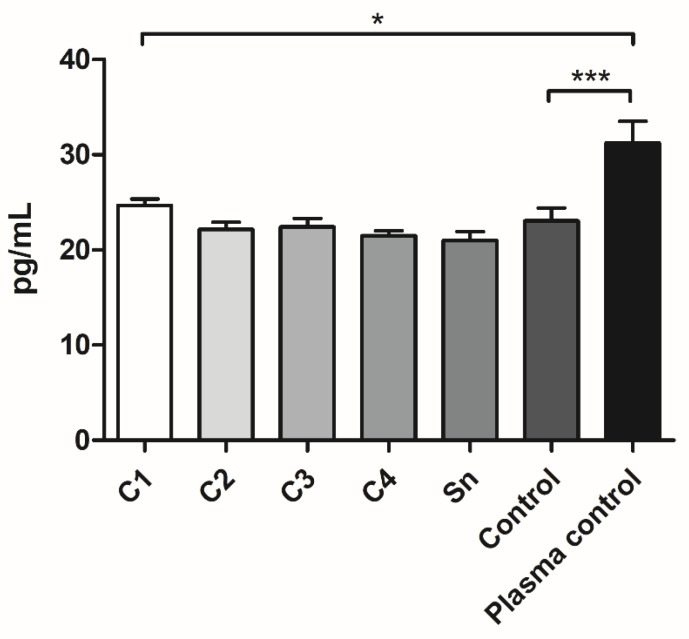
The PDGF-AB concentration of the FFP samples: different cryoprecipitate groups, supernatant (Sn), and fresh frozen plasma control (Control), where the cryoprecipitate was resolubilized in 10 mL (C1), 20 mL (C2), 30 mL (C3), and 40 mL (C4) plasma (*n* = 8: the FFP derived from four different donors, and the samples were measured in duplicate) and of the plasma samples, which were isolated manually (Plasma control, *n* = 10, the samples were taken from five donors and measured in duplicate). The significance level was *p* < 0.05, where * means that *p* is between 0.01 and 0.05, ** means that *p* is between 0.01 and 0.001, and *** means that *p* is lower than 0.001, and data are presented as mean ± standard error of the mean.

According to the PDGF-AB measurement, a slight and not significant tendency could be observed in the cryoprecipitate samples and supernatant, showing that more concentrated cryoprecipitates contain more PDGF-AB. The FFP control contained more PDGF-AB than C2, C3, C4, and the supernatant. Manually isolated plasma contained significantly higher PDGF-AB concentrations than all the FFP samples (Figure 5).

### 3.4. Live-Dead Staining of hBM-dMSCs Cultured on the Fibrin Membranes

**Figure 6:**
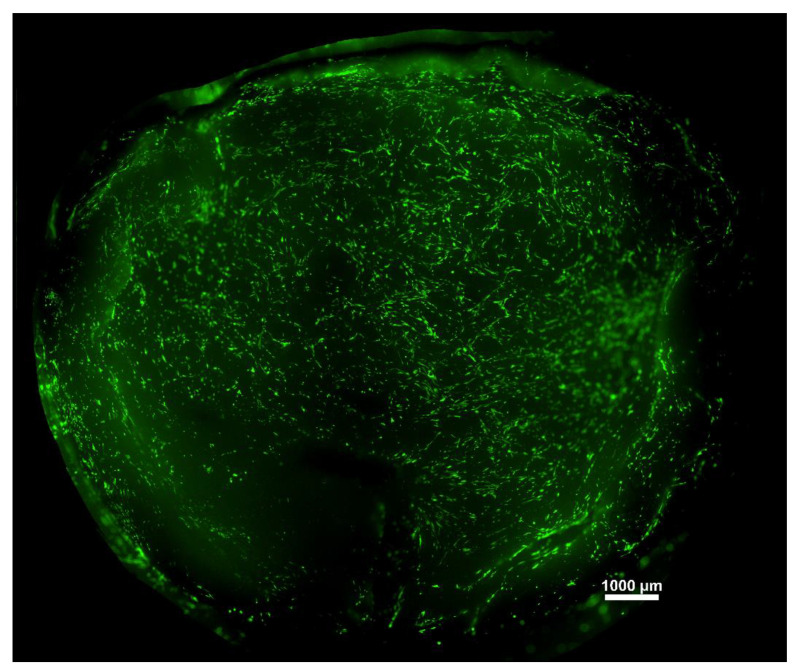
A representative image of hBM-dMSCs stained with Calcein-AM attached to a fibrin membrane.

Live-dead staining was conducted to visualize the cells cultured on the membranes for seven days. A representative large-scale image was created to have an overview of the hBM-dMSCs growing on the scaffolds (Figure 6).

**Figure 7:**
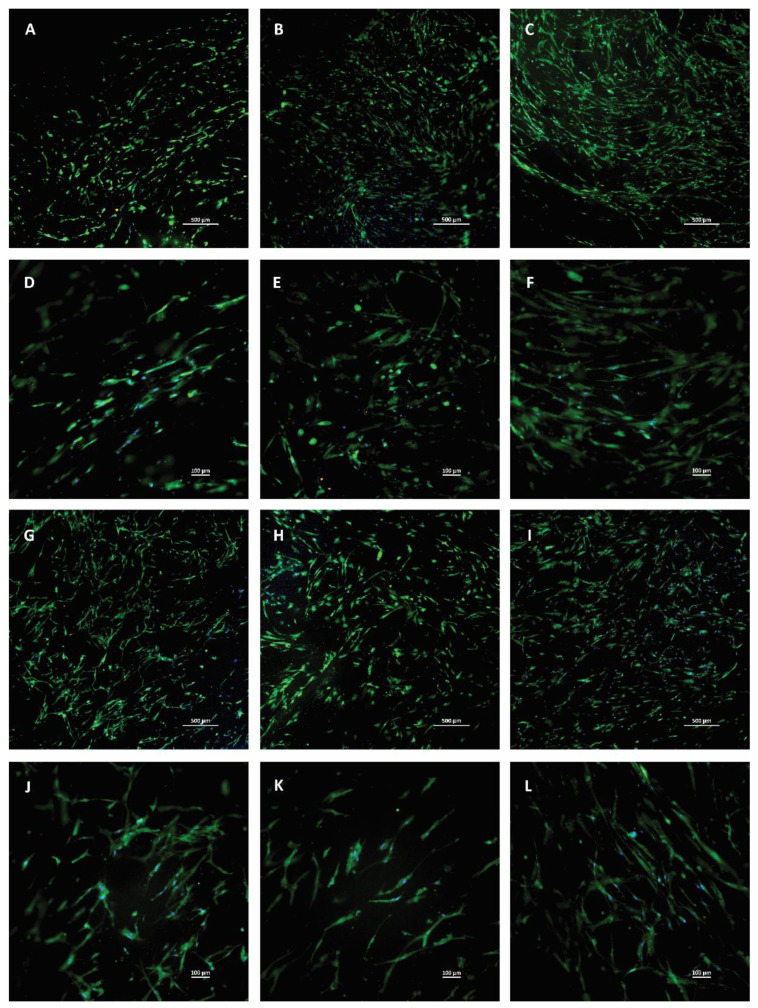
Live-dead staining of hBM-dMSCs cultured on fibrin membranes with different thicknesses. Living cells are green, dead cells are yellow, and nuclei are blue. (**A**–**C**) and (**G**–**I**) were taken in 4× magnification, and the scale bar represents 500 µm, (**D**–**F**) and (**J**–**L**) were taken in 10× magnification and the scale bar represents 100 µm. The cells were seeded on membranes isolated from cryoprecipitate, which was dissolved in 10 mL (C1, parts **A** and **D**), 20 mL (C2, parts **B** and **E**), 30 mL (C3, parts **C** and **F**), and 40 mL (C4, parts **G** and **J**) plasma, from supernatant (Sn, parts **H** and **K**), which collected from above the cryoprecipitates, and pooled, and from plasma, which was used as a control (parts **I** and **L**).

More detailed images were taken of the individual membranes with 4× and 10× magnification to see the attached cells. Living cells are stained with green, dead cells are yellow, and nuclei are blue. A few dead cells could be observed among living cells; however, their number was minimal. The distribution of the cells was not homogenous, as there were preferred regions with more cells, which can be seen in the images. However, based on the microscopic images, the thickness of the membrane does not have any effect on the viability and differentiation of the hBM-dMSCs; no tendency can be observed (Figure 7).

### 3.5. Viability of hBM-dMSCs Cultured on the Fibrin Membranes Measured by XTT

**Figure 8:**
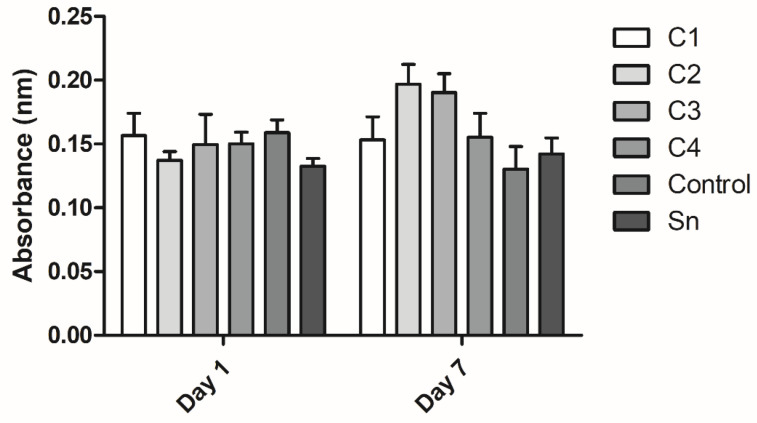
The viability of human mesenchymal stem cells cultured on the fibrin membranes. Cell attachment was examined on the first day and proliferation was examined after 7 days of culturing. The membranes were isolated from cryoprecipitate, which was resolubilized in 10 mL (C1), 20 mL (C2), 30 mL (C3), and 40 mL (C4) plasma, from supernatant (Sn), which was collected from above the cryoprecipitate and pooled, and from plasma, which was used as a control (*n* = 3 on day 1 and *n* = 4 on day 7).

XTT measurement was conducted to examine cell viability on the membranes on the first and seventh days. On the first day viability was measured to obtain information about the cell adhesion onto different membranes. No significant difference was observed in the cell attachment (Figure 8).

On the seventh day the proliferation of the cells was examined on the membranes. The differences were not significant, however, C2, C3, C4, and the control groups showed a growing tendency in cell attachment with the membrane thickness. The C1 and supernatant groups did not follow this trend. The difference in cell viability between the first and the seventh days was not significant either (Figure 8).

## 4. Discussion

Although the used plasma was prepared by plasmapheresis, it still contained a small amount of cellular elements and their concentration was directly proportional to the cryoprecipitate concentration. The presence of leukocytes and red blood cells in the samples may only be due to the centrifugation, not because of the solvent properties, as they were not reported to be enriched in cryoprecipitate. The concentration of platelets also increased with the increasing fibrinogen content of cryoprecipitates, and in this case the differences were significant. However, this may also be due to the centrifugal separation, not because of the reduced solubility at low temperatures. The platelet concentration is important because upon activation they release platelet-derived growth factors to their environment, which may be entrapped in the fibrin network, and thus promote regeneration. However, the platelet concentration of plasma isolated by plasmapheresis is lower than manually isolated PPP [27]: using cryoprecipitate this drawback can be evaded.

From our point of view the most important component of cryoprecipitate was fibrinogen. Our measurements revealed the increasing concentration of fibrinogen, when the cryoprecipitate was dissolved in decreasing amounts of plasma, and most of the differences were significant. The fibrinogen concentration influences the thickness of the membranes, which can be prepared from cryoprecipitate or plasma by activation.

The total protein content was slightly, but significantly affected by cryoprecipitate isolation. It may be because of fibrinogen and other proteins with low solubility at low temperatures. Albumin also showed a mild decreasing tendency with decreasing cryoprecipitate concentrations despite its good water-solubility, and ALP enzyme activity was also lower in the supernatant than in cryoprecipitates, but the difference was not significant.

A small amount of immunoglobulins can be found in cryoprecipitates [5], and we also observed a mild difference in IgG and IgM concentrations between cryoprecipitates and supernatants; however, the difference was not significant.

The supernatants belonging to different cryoprecipitates showed similar properties, thus they were pooled in our later experiments.

According to our hypothesis, the freeze-dried weights of the membranes were greater if they were isolated from more concentrated fibrinogen solutions. The membranes were isolated in 24-well cell culture plates, thus their diameter was identical, but their thickness increased with increasing cryoprecipitate concentrations.

The PDGF-AB measurement showed that manually isolated plasma contains significantly more growth factors than each sample derived from FFP, which supported our expectation, regarding that FFP was isolated by plasmapheresis, which removes most of the platelets. However, according to the platelet measurement (Figure 1A), the platelet number of C1 was comparable to the manually isolated PPP, which contains 34.5 × 10^9^/L [27]. Besides, the control, which was also FFP, contained more PDGF-AB than C2, C3, and C4. Thus, it seems, that cryoprecipitate isolation may cause the partial degradation of PDGF-AB. Between cryoprecipitate samples a slight tendency can be observed indicating that a thicker membrane might contain more growth factors, thus promoting more cell attachment and regeneration.

As it was reported previously [25], hBM-dMSCs attach onto fibrin membranes due to their biocompatible structure. Live-dead staining and microscopic analysis did not show any visible difference between the membranes in cell attachment capacity despite the presumable higher platelet and growth factor concentration of thicker membranes.

The XTT measurement showed that the difference between cell attachment and proliferation on the different membranes was not significant. The similarity of cell viability between day 1 and day 7 shows that the proliferation of the cells was slow and weak; however, the number of the cells did not shrink, indicating that the membranes were not cytotoxic. The anticoagulant and Ca-gluconate present in the activated FFP may cause a less favorable environment than PRF, which does not contain any anticoagulant, therefore no activation is needed and it promotes the adhesion and proliferation of hBM-dMSCs [25].

The tendency in cell viability on the seventh day may be due to the higher growth factor concentration in the thicker membranes; however, cell viability on C1 was lower than on C2 and C3. The anticoagulant and Ca-gluconate concentration might also be higher in thicker membranes, causing an adverse effect in cell viability.

## 5. Conclusions

Based on our results we concluded that cryoprecipitate isolation is a working method for preparing plasma products with increased fibrinogen and platelet concentration, while the level of the other components remains roughly similar. The PDGF-AB level increases slightly in more concentrated plasma samples because of the higher platelet number. The membranes isolated from different plasma samples are getting thicker with increasing fibrinogen concentration, which can result in improved application in tissue engineering. The slight increase in the PDGF-AB concentration did not cause a significant increase in cell proliferation on thicker membranes. Membranes isolated from FFP were found to have a lower cell attachment and proliferation-promoting effect than PRF was reported to possess, probably because of the added anticoagulant and Ca-gluconate needed for activation. However, the advantage of FFP is its stability due to the added anticoagulants, while PRF must be isolated directly after blood drawing [30], making its use more difficult in regenerative medicine. It must be noted that due to the antibody content of these membranes, similarly to PRF membranes, the blood products can only be used between donors and recipients with matching AB0 and Rh blood types or autologously [21]. The applicability of plasma-derived membranes in vivo, for example as wound dressings, is yet to be examined, besides, membranes isolated from purified fibrinogen instead of cryoprecipitate can be another interesting topic. According to our experiments these membranes may be the base of a new innovative product for tissue engineering.

## Data Availability

The data presented in this study are available on request from the corresponding author.
